# β-*N*-Methylamino-l-alanine (BMAA) Toxicity Is Gender and Exposure-Age Dependent in Rats

**DOI:** 10.3390/toxins10010016

**Published:** 2017-12-27

**Authors:** Laura Louise Scott, Timothy Grant Downing

**Affiliations:** Department of Biochemistry and Microbiology, Nelson Mandela University, P.O. Box 77 000, Port Elizabeth 6031, South Africa

**Keywords:** β-*N*-methylamino-l-alanine, BMAA, rats, behavior, neurodegeneration, rat, cognition, motor function

## Abstract

Cyanobacterial β-*N*-methylamino-l-alanine (BMAA) has been suggested as a causative or contributory factor in the development of several neurodegenerative diseases. However, no BMAA animal model has adequately shown clinical or behavioral symptoms that correspond to those seen in either Alzheimer’s Disease (AD), Amyotrophic Lateral Sclerosis (ALS) or Parkinson’s Disease (PD). We present here the first data that show that when neonatal rats were exposed to BMAA on postnatal days 3, 4 and 5, but not on gestational day 14 or postnatally on days 7 or 10, several AD and/or PD-related behavioral, locomotor and cognitive deficits developed. Male rats exhibited severe unilateral hindlimb splay while whole body tremors could be observed in exposed female rats. BMAA-exposed rats failed to identify and discriminate a learned odor, an early non-motor symptom of PD, and exhibited decreased locomotor activity, decreased exploration and increased anxiety in the open field test. Alterations were also observed in the rats’ natural passive defense mechanism, and potential memory deficits and changes to the rat’s natural height avoidance behavior could be observed as early as PND 30. Spatial learning, short-term working, reference and long-term memory were also impaired in 90-day-old rats that had been exposed to a single dose of BMAA on PND 3–7. These data suggest that BMAA is a developmental neurotoxin, with specific target areas in the brain and spinal cord.

## 1. Introduction

The cyanobacterial metabolite β-*N*-methylamino-l-alanine (BMAA), found in many commonly consumed food items [1,2,3,4,5,6,7,8,9,10,11,12,13], has been implicated in the development of the neurodegenerative diseases Amyotrophic Lateral Sclerosis/Parkinsonism Dementia Complex (ALS/PDC) [14], Amyotrophic Lateral Sclerosis (ALS) and Alzheimer’s Disease (AD) [15]. This proposed link is based largely on the high concentration of the amino acid in the diet of the Chamorro people of Guam, who had an extremely high incidence of ALS/PDC, and on the detection of BMAA in the brain tissue of Chamorro ALS/PDC, Canadian AD, sporadic ALS and Parkinson’s Disease (PD) patients. However, to date no animal model of BMAA toxicity has successfully shown clinical or behavioral symptoms together with neuropathology, that correspond to symptoms observed in patients with these neurodegenerative diseases. 

Although acute toxicity of BMAA, when administered at doses as high as 4000 mg per kilogram body weight [16], has been shown in chicks [17], rats, and mice [16,18,19,20,21] and some neurotoxic effects such as hind leg splay and rigidity have been described when BMAA is administered via intracerebroventricular injections [22,23], studies using systemic administration of an environmentally relevant BMAA dose to adult animals have not been successful in linking BMAA to neurodegenerative diseases. Polsky et al. [18], Perry et al. [24], Duncan et al. [25], Cruz-Aguado et al. [26] and Scott et al. [27] did not observe any clinical signs of toxicity associated with the chronic administration of BMAA to adult rats, and only Seawright et al. [16] were able to show some neurodegeneration with chronic high dose exposure (1000 mg BMAA/kg body weight per day). Karlsson et al. [20] attributed the lack of symptoms observed in adult rats to the low transport of BMAA into the adult rodent brain [20,25,28] and subsequently showed a substantially higher transfer of BMAA into the brain of neonatal rats with distinct localization of the neurotoxin in the hippocampus and striatum. Additionally, Smith et al. [28] demonstrated that BMAA gains access to the brain via the cerebrovascular large neutral amino acid transporter and consequently suggested that modulators of brain neutral amino acid uptake, such as the observed enhanced uptake rate in infants [29,30], would similarly modulate the transport of BMAA into the brain. 

Following BMAA exposure on postnatal day (PND) 2 and/or PND 5 Dawson et al. [31] observed reduced cerebellar weights in male and female rats and neurochemical changes in BMAA-exposed male rats, but not in exposed female rats. These changes, that are indicative of alterations in the development of the spinal cord and cerebellum, were accompanied by increased hind leg splay in male and female BMAA-exposed rats and a mild elevation in the blood pressure in female rats. This study by Dawson et al. [31] is the only report of gender differences associated with BMAA toxicity. Although both Dawson et al. [31] and Karlsson et al. [19,20,32,33] tested the effect of BMAA on neonatal rats, Dawson et al. [31] did not conduct full behavioral, emotional and cognitive evaluations and Karlsson et al. [19,20,32,33] only did PND 9 and PND 10 BMAA exposures. Karlsson et al. [19,20,32] reported that exposure to 600 mg BMAA/kg on PND 9 and 10 may lead to some mild disturbances in motor function and failure to habituate to a novel test environment and that early exposure may result in cognitive impairments in adulthood, but were unable to fully correlate the observed behavioral deficits to symptoms typically observed in ALS/PDC, AD and/or PD patients. Karlsson et al. [19,20,32] were also unable to replicate some of the observations reported by Dawson et al. [31] and attributed this to the different exposure ages used and specifically indicated that the exposure age (PND 9–10) falls within the brain growth spurt (BGS). Interestingly, Dobbing and Sands [34] reported that the BGS, defined as the total brain weight gain as a percentage of the adult weight, peaks at PND 7 in rats, and not at PND 10, while Gottlieb [35] reported that an initial rapid increase in dendrite density and brain complexity occurs between PND 0–6, as was also observed by Baloch et al. [36] and Bockhorst et al. [37], causing Semple et al. [38] to suggest that developmental toxicity studies should, as a rule of thumb, be conducted on rats aged 4–7 days. Furthermore, although Karlsson et al. [20] have demonstrated the transplacental transfer of BMAA in rats, behavioral studies and neuropathology that support the toxicity of *in utero* BMAA exposure are lacking. 

The observation that neonatal and adult rats respond differently to BMAA, together with the findings of epidemiological studies conducted by Garrutto et al. [39] and Sabel et al. [40] showing that exposure to environmental factors *in utero* or in the early stages of life, may be important for the development of ALS several years later, suggests that age of exposure might be the determining factor of BMAA neurotoxicity.

Reported deficits or behavioral abnormalities observed in BMAA-exposed animals or in other accepted animal models of AD and PD, have thus far failed to match all of the deficits that are commonly seen in patients with AD, PD or ALS. 6-OHDA results in impaired motor coordination and decreased locomotion of rodents in the open field test, but data on anxiety-like behavior, mood changes, visuo-spatial, long-term and working memory deficits, all symptoms frequently associated with PD, remain inconclusive [41]. Similarly, the MPTP mouse model fails to encompass the wide assortment of motor impairments seen in PD patients [42]. Rotenone exposure, on the other hand, does result in most PD-like motor symptoms and neuropathology, but seems to improve rather than impair spatial learning and memory abilities in treated mice [42]. There also appears to be an intrinsic resistance of some rats to rotenone, and as few as 50% of treated animals within an experimental group exhibits neurodegeneration [43] and in the rats that do respond to rotenone, the time span during which the individual rats develop the symptoms, varies considerably [44]. Without a suitable animal model for AD, PD and ALS/PDC it is impossible to investigate disease mechanisms or potential therapies, or conduct preclinical studies.

Knowledge of the effects of exposure to BMAA at different developmental stages remains limited and there is no report that compares the potential sex-dependent BMAA-induced alterations in behavioral, emotional and cognitive responses following *in utero* exposure, or exposure at critical stages of neonatal brain development. We therefore sought to investigate the effect, measured by standard rodent behavioral and cognitive response tests, of BMAA on male and female rats when exposed to the neurotoxin on gestational (G) day 14, PND 3, 4, 5, 6, 7 and 10 and from this potentially develop the first rodent model for ALS/PDC. These exposure times were selected to target specific developmental stages in the rodent brain so as to identify potential brain regions and/or processes involved in BMAA toxicity. BMAA exposure on G 14 aimed to target the developing brain just before the onset of formation of the dentate gyrus (G 18) and hippocampal regions (G 15.5) [45,46,47]. Postnatal BMAA exposure, conversely, was intended to target hippocampal and dentate gyrus neurogenesis which peaks at PND 3, 5 and 5, with 80% of cells being produced at this age [48,49] and substantially decreases at the start of PND 7 [48]. Neurogenesis forms the basis for the normal structure and function of the adult brain and plays a critical role in the formation of hippocampal-dependent spatial learning and memory function later in life. Additionally, at PND 0–2, the first distinct patches of dopamine fibers are distributed throughout the striatum after which dopaminergic innervation increases substantially throughout the developing brain [50,51] Dopamine and serotonin modulate several aspects of neuronal development, including cell proliferation, migration and differentiation, and furthermore contribute to the development of pathways needed for movement, cognition and reward [52,53,54,55,56] Altered dopaminergic and serotonergic signaling during development can therefore produce long lasting changes that contribute to neuropsychiatric and neurodegenerative disorders. 

## 2. Results and Discussion

### 2.1. General Findings

All pups were closely monitored for seizure and/or abnormal behavior after subcutaneous injection with 400 mg/kg BMAA. Although Karlsson et al. [20] observed delayed motor convulsions and emprosthotonic-like seizures in some animals following administration of 650 mg/kg, no acute adverse effects were observed following BMAA administration at a dose 400 mg/kg, and no weight changes were observed in the BMAA-treated rats compared to the control and vehicle control groups over the 4-month test period.

The PND 3, 4 and 5 BMAA-exposed male rats exhibited pronounced hind leg splay and hind leg rigidity similar to that observed by Dawson et al. [31] following BMAA administration to male rats on PND 2 and PND 5 and by Rakonczay et al. [22] after ICV BMAA administration to adult male rats. Interestingly, Goldstein et al. [57] also observed hind leg splay and rigidity in rats following administration of a vesicular monoamine transporter (VMAT2) inhibitor, reserpine, which could be attenuated by administration of centrally acting dopaminergic and anti-cholinergic drugs. Lower body tremors were observed in female BMAA-exposed rats. The hind leg splay in males and tremors observed in female rats were however transient, disappearing by PND 45 and PND 30 respectively. Additionally, male and female rats exposed to BMAA on PND 3, 4 or 5 had heightened anxiety compared to control rats and exhibited a low or flat body posture and dragging of the tail. Increased anxiety and low body posture in rats have typically been associated with a behavioral syndrome brought on by administration of monoamine oxidase inhibitors [58] and also by exposure to 3,4-methylenedioxymethamphetamine (MDMA), a monoamine reuptake inhibitor and VMAT inhibitor, to rats. [59,60]. The reported increased anxiety in PND 3, 4 and 5 exposed rats (400 mg/kg BMAA) described here is based on the observed increased defecation, a significant indicator of anxiety, in the open field test (data not shown) but is also confirmed by the increased thigmotaxic behavior observed in rats in several conducted behavioral tests as discussed below. 

**Figure 1:**
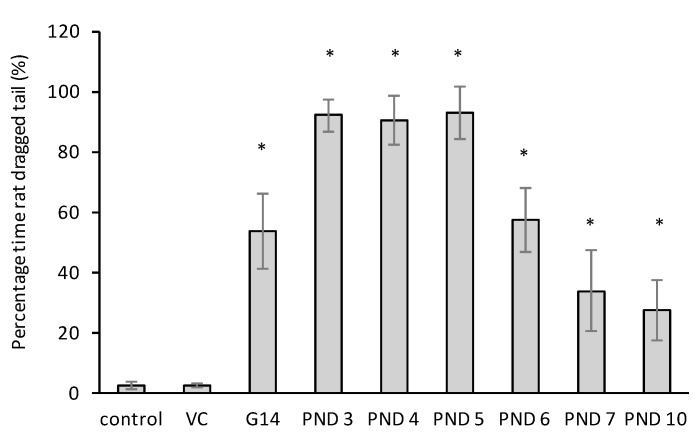
Percentage time rat dragged its tail, as observed in male and female Sprague Dawley rats (*n* = 30 for vehicle control and *n* = 10 for each other exposure age as indicated on the figure) during a ten-minute test period on PND 18. * Indicates significant difference to the control (*p* < 0.05).

Tail dragging, a sign of spinal cord damage and/or degeneration, was observed in all male and female BMAA-exposed rats (Figure 1). Hamers et al. [61] demonstrated that in rats, tail dragging is a symptom of contusion injury that can usually be observed up to 4 weeks following injury and one week following transection injury of the dorsal column. In both these models described by Hamers et al. [61] the number of animals exhibiting tail drags and the mean duration thereof decreased over time, similar to what was observed in rats neonatally exposed to BMAA in this study, where tail dragging could be observed in male and female rats during the nestfinding test on PND 10 and in the open field test on PND 20 but not in the elevated plus maze on PND 30.

Eye opening, a developmental landmark, was delayed by two days in four out of five female rats and all male rats treated with BMAA on PND 5 and in all the male and female rats treated with BMAA on PND 3 or 4. Eye opening is related to the serotoninergic and dopaminergic system, and its delay is considered a significant indicator of reduced dopamine and/or serotonin in the brain [62,63]. It is interesting to note that the other gross clinical deficits observed in BMAA-exposed rats in this study are also, for the most part, comparable to those observed in rats that have been exposed to agents that interfere with dopaminergic and/or serotonergic transmission.

### 2.2. The Nest Finding Test

**Figure 2:**
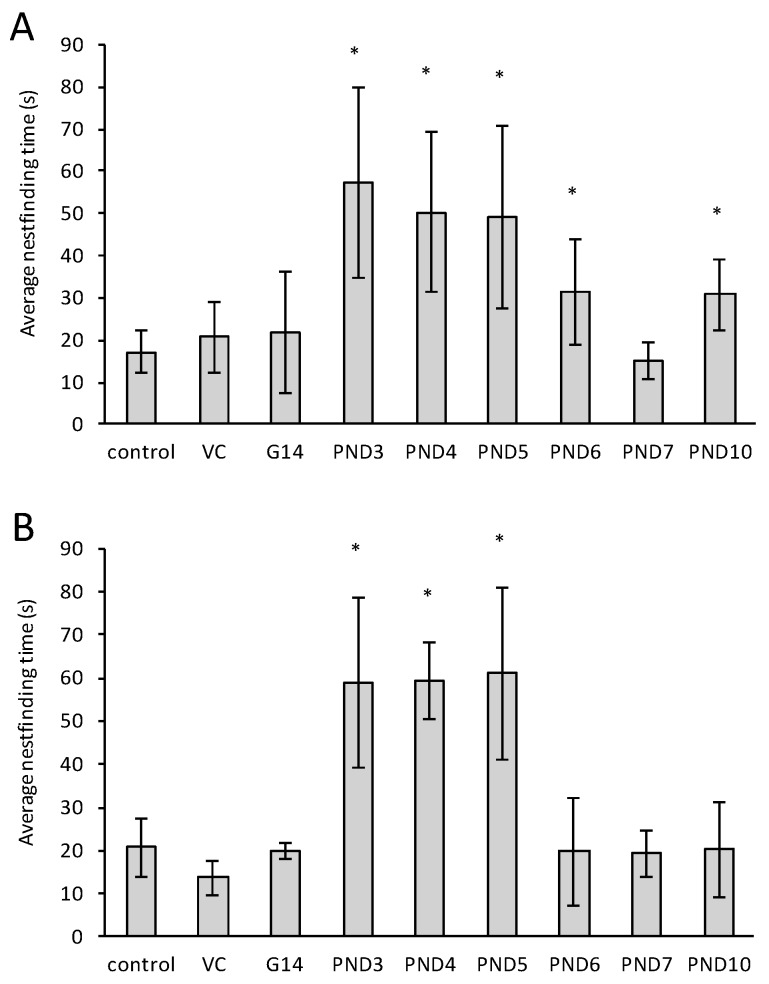
Average nest finding times of male (**A**) and female (**B**) Sprague Dawley rats tested on PND 10. VC is vehicle control, G14 is exposure on gestation day 14. For the vehicle control *n* = 15 whereas *n* = 5 for all other groups. * Indicates significant difference to the control (*p* < 0.05).

The nest finding test monitors olfaction and odor discrimination in a neonatal rat by evaluating the rat’s ability to discriminate the smell of unfamiliar clean bedding, from that of its own home cage nest which it will preferentially try to reach [64]. Figure 2 shows the results of nest finding times for control rats as well as rats exposed *in utero*, and up to postnatal day 10. All the control pups located their home nest and fulfilled the criteria for a positive response. Maximum effect on olfactory function and/or odor discrimination ability was observed in PND 3, 4 and 5 exposed male (Figure 2A) and female (Figure 2B) rats with animals in these groups taking, on average, more than three times longer to reach their nests than control rats. A lesser, but still significant, effect was observed in males exposed to BMAA on PND 6 and 10 and not in female rats exposed at the same ages. There were no significant differences between control group rats and female rats exposed to BMAA prenatally, or on PND 6, 7 or 10, or male rats exposed prenatally or on PND 7. Cumulative doses of G 14, PND 5 and 10 were not significantly different from PND 5 (data not shown), with the effects being attributed to PND 5 exposure. This confirms that age of exposure is more important than total perinatal dose. Another general finding was that male and female pups exposed to BMAA on PND 3, 4 and 5 appeared to be hypoactive and had an unsteady gait together with a flat body posture as discussed in the general finding section.

The longer average nest finding time required by BMAA-exposed rats was limited by a maximum test duration, and thus score, of 90 s. However, several of the rats from the exposed groups did not reach the correct nest during this time. Based on observations this was attributed to loss of olfactory discrimination and not reduced locomotor ability. Table 1 lists the percentage of rats that reached the correct nest in each of the three trials for the various treatment groups. Hyposmia and lack of odor identification and/or discrimination, as observed in rats neonatally exposed to BMAA, are typical non-motor symptoms of early stages of PD [65,66] and AD in humans [67]. Difficulty in recognizing and recalling the odor of a home nest compared to an unfamiliar nest, which is a learned response, suggests that BMAA potentially damages the hippocampus and dentate gyrus, regions involved in cognitive or memory processing. Bohnen et al. [68] demonstrated that selective hyposmia in PD is most robustly correlated with a reduction in mesolimbic hippocampal dopamine innervation and concluded that damage to the hippocampus specifically, is a significant determinant of lack of odor discrimination in PD. 

### 2.3. The Modified Open Field Test

**Figure 3:**
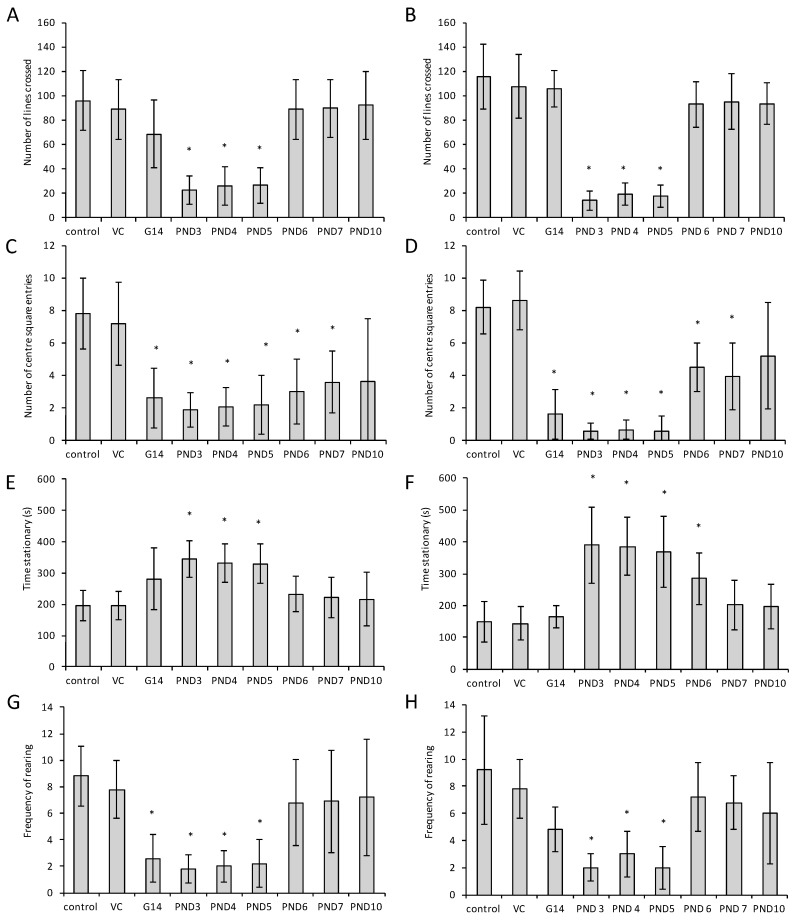
Results of the modified Open Field Test in male (**A**,**C**,**E**,**G**) and female (**B**,**D**,**F**,**H**) Sprague Dawley rats. (**A**,**B**) shows the total number of lines crossed, (**C**,**D**)shows the number of entries into the center squares. (**E**,**F**) shows the total stationary time and (**G**,**H**) the number of rearing events. For each dose age and gender group *n* = 5, except for the vehicle control where *n* = 15. Significance (*p* < 0.05) is indicated by (*).

Locomotor activity, measured by the number of lines crossed (Figure 3A,B) and the time stationary (Figure 3E,F) are significantly (*p* < 0.05) reduced as a function of BMAA exposure on PND 3, 4 and 5, with a slightly greater effect observed in female rats. Increased anxiety can be seen in rats exposed to BMAA from G 14, and postnatally in all exposure groups up to PND 7 in both male and female rats. This is apparent from the increased thigmotaxic behavior (Figure 3C,D) and increased defecation observed (data not shown). In humans, increased anxiety is typically observed in patients with early AD [69] and PD [70]. Rearing was also significantly reduced in rats exposed to BMAA on PND 3, 4 and 5 and suggests (Figure 3G,H), together with the observed low locomotor activity, that these rats also exhibit less spontaneous exploratory activity in a novel environment. This could either be the result of failure to detect spatial novelty, which is dependent on both the dentate gyrus and the CA3 region of the hippocampus [71], or to lack of motivation to explore, which is mediated mainly by the dentate gyrus [72]. Lever et al. [73] and Saab et al. [72] reported that upon novelty detection the nucleus accumbens, a region responsible for initiating rearing [73] receives input from the dentate gyrus *via* either the trisynaptic hippocampal loop or through the entorhinal cortex and the subiculum. Thus, the observed decrease in rearing suggests BMAA-induced damage of the dentate gyrus and/or the hippocampus. Similar reductions in exploration and/or novel location recognition was observed in male and female rats neonatally exposed to methamphetamine, a drug that elicits its effect by triggering a massive release of dopamine (DA) due to binding to the dopamine reuptake transporter (DAT), by displacing vesicles and by the inhibition of monoamine oxidase, and by enhancing the DAT-mediated reverse transport of DA transport across the plasma membrane [74]. The number of observed rearings in the open field test, and thus exploration, is, in addition to being a result of hippocampal damage, also strongly dependent on dopaminergic and GABAergic control [75,76], with dopamine agonists enhancing the number of rears [77] and GABAergic agonists having the opposite effect [78,79]. Based on the open field results it is thus not unlikely that BMAA could, as with methamphetamine, exert its effect by interfering with the brain dopaminergic system in rats. Interestingly, epidemiological studies have shown that methamphetamine users have two times the risk of developing PD compared to non-users [80] while, additionally, a causal link has been suggested between methamphetamine use and development of ALS later in life [81], and development of brain damage similar to that observed in Alzheimer’s Disease patients is seen in animals exposed to methamphetamine [82]. Thus, since BMAA and methamphetamine exposure are both associated with the delayed onset of the same neurodegenerative diseases and exert the same behavioral and cognitive effects following neonatal exposure in rodents it is plausible that BMAA might be a specific, although not selective, dopaminergic toxin.

### 2.4. The Elevated Plus Maze

The elevated plus maze (EPM) is a simple method for assessing the anxiety responses of rodents by relying on a rodent’s proclivity to enclosed spaces and its natural avoidance of heights and open spaces [83]. 

**Figure 4:**
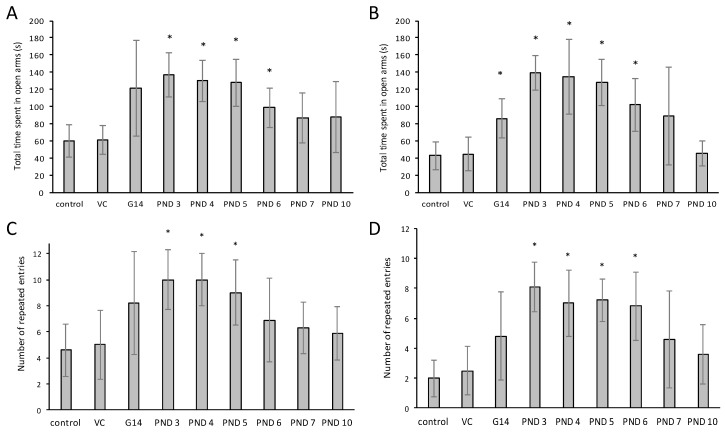
Average time spent in the open arms of the elevated plus maze observed in male (**A**) and female (**B**) Sprague Dawley rats and the number of repeated entries made by male (**C**) and female (**D**) rats into the open arms during the 10-min testing period as tested on PND 30. For the vehicle control *n* = 15, and *n* = 5 for all other groups as indicated on the graphs. * Indicates significant difference from the control (*p* < 0.05).

Control and vehicle control rats exhibited normal behavior in the elevated plus maze, with limited initial open arm exploration and subsequent entry into a protected arm where they spent the majority of the remaining test time [83]. Male and female rats (Figure 4) exposed to BMAA on PND 3, 4, 5 and 6, and female rats exposed to BMAA *in utero*, spent a significantly longer total time on the open arms compared to the control rats. Karlsson et al. [19] also reported that rats exposed to 600 mg/kg BMAA on PND 9 and 10 spent significantly more time on the open arms of the maze. Increased activity on the open arms is generally considered to be an indication of reduced anxiety. In our study, however, exposed rats did not spend significantly more time on the open arms per visit, but rather repeatedly entered the open arms (Figure 4C,D) which resulted in an overall higher total time spent (Figure 4A,B) compared to the control rats. Greater variations in the average time spent on the open arms per visit were observed between females of the same group compared to that of males. Marcondes et al. [84] studied the influence of estrous cycle on open arm activity in the elevated plus maze and reported that rodents in proestrus spend more time on the open arms of the elevated plus maze compared to rodents in diestrus. Our data suggest that the apparent decreased anxiety (increase in total time spent and/or higher number of repeated entries into the open arms) was probably due to the short-term memory impairment observed (see below) and not due to reduced anxiety, as anxiety levels were higher as measured in the open field test. Alternatively, since the EPM was conducted 10 days after the open field test, anxiety may have been reduced by this time along with certain other symptoms as described above.

### 2.5. The Inclined Plane Test

The inclined plane test done on PND 37 showed no effect of exposure to 400 mg/kg BMAA on either G14, PND 3, 4, 5, 6, 7 or 10. Karlsson et al. [19] also reported no effect in rats exposed to 650 mg/kg BMAA on PND 9–10.

### 2.6. Audiogenic Response

**Figure 5:**
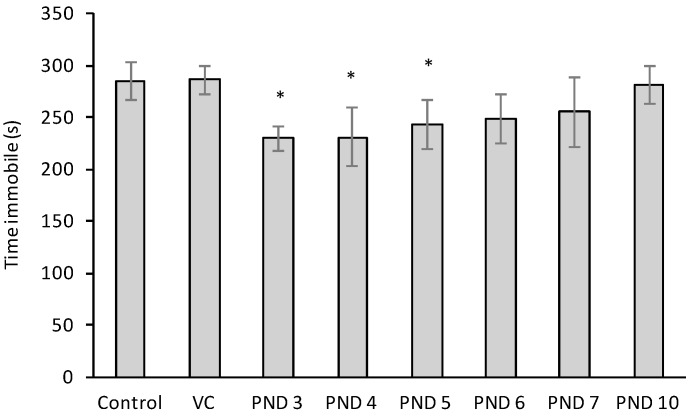
Duration Sprague Dawley rats (*n* = 30 for vehicle control and *n* = 10 for all other groups) were immobile after a 120 dB noise, following 5-min habituation to the test environment, as tested on PND 55 in the audiogenic freezing response test. * Indicates significant difference from the control (*p* < 0.05).

The audiogenic freezing test monitors a passive defense mechanism in rats that reaches adults levels at 40 days of age [85]. The duration of the freezing response, following an unexpected 120 dB noise, was significantly reduced in rats injected with 400 mg/kg BMAA on PND 3, 4 and 5 and only slightly, but not significantly, reduced in rats that was exposed to BMAA on PND 6 and 7 (Figure 5). Karlsson et al. [19] also reported a reduced freezing time in rats injected with 600 mg/kg BMAA on PND 10 whereas Hard et al. [86] observed the same reduction in rats that received intracisternal injections of 6-OHDA neonatally. Hard et al. [85,86] suggested that interference with the developing serotoninergic and dopaminergic systems can influence this defense reaction, but the complete neurobiological mechanisms remain unknown.

### 2.7. The Radial Arm 

Working and reference memory are typically assessed using the eight-arm land-based radial arm maze. Optimally, the animal should learn to visit only the always-baited arms and never the always-unbaited arms. Figures 7 and 8 show the substantial effect of BMAA on reference memory and learning as observed in male and female rats, respectively, and Figure 9 the effect of BMAA on working memory.

**Figure 6:**
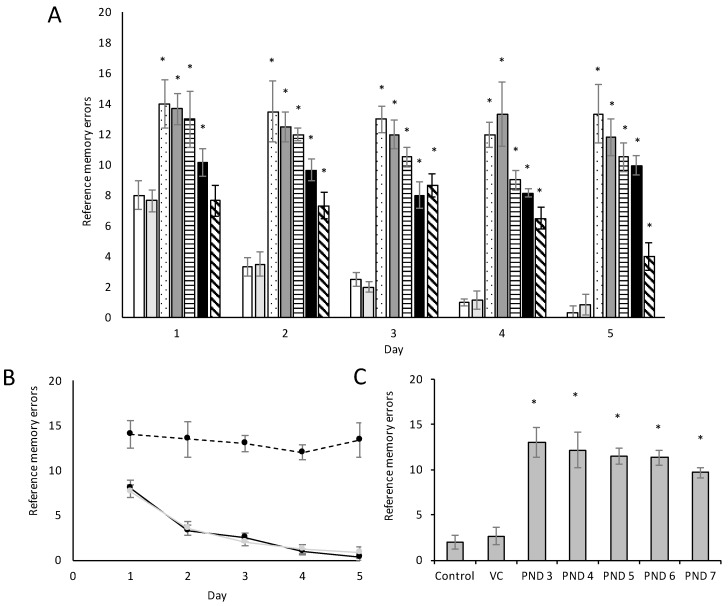
Reference memory errors observed in male Sprague Dawley rats (*n* = 15 for vehicle control and *n* = 5 for all other groups) exposed to BMAA on PND 3 (dotted bar), PND 4 (dark grey bar), PND 5 (horizontally striped bar), PND 6 (black bars) and PND 7 (diagonally striped-bars) compared to control (white bars) and vehicle control rats (light grey bars) in the acquisition trials (**A**) of the radial arm maze. In normal, healthy rats the observed reference memory errors will decrease with each consecutive acquisition trial as a function of learning. (**B**) Indicates the change in reference memory errors, or learning, over the five-day trial period observed in control (black line), vehicle control (grey line) and rats exposed to BMAA on PND 3 (black dashed line). Long-term memory, or retention of the learned task, allows the rats to remember the learned pattern on the radial arm maze. The reference memory errors observed in the retention test of the radial arm maze conducted one week after the last acquisition trial (**C**) compares the long-term memory of rats exposed to BMAA on PND 3–PND 7 compared to control rats. (*) Indicates significant difference from the control (*p* < 0.05).

**Figure 7:**
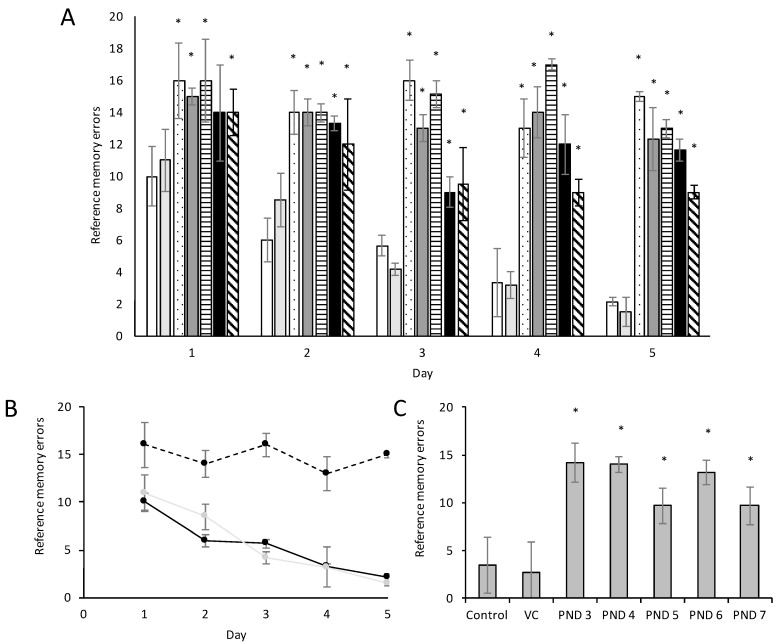
Reference memory errors observed in female Sprague Dawley rats (*n* = 15 for vehicle control and *n* = 5 for all other groups) exposed to BMAA on PND 3 (dotted bars), PND 4 (dark grey bars), PND 5 (horizontally striped-bars), PND 6 (black bars) and PND 7 (diagonally striped-bars) compared to control (white bars) and vehicle control rats (light grey bars) in the acquisition trials (**A**) of the radial arm maze. In normal, healthy rats the observed reference memory errors will decrease with each consecutive acquisition trial as a function of learning. (**B**) Indicates the change in reference memory errors, or learning, over the five-day trial period observed in control (black solid line), vehicle control (grey line) and rats exposed to BMAA on PND 3 (black dashed line). Long-term memory, or retention of the learned task, allows the rats to remember the learned pattern on the radial arm maze. The reference memory errors observed in the retention test of the radial arm maze conducted one week after the last acquisition trial (**C**) compares the long-term memory of rats exposed to BMAA on PND 3–PND 7 compared to control rats. (*) Indicates significant difference from the control (*p* < 0.05).

The five-day acquisition period revealed significant differences between the groups. The number of reference memory errors, defined as the entry into arms that have never been baited and thus a test of the rat’s recollection of the previous trials, decreased by roughly 90% from acquisition day 1 to acquisition day 5 in male control and vehicle control groups, but decreased by only 6% from acquisition day 1 to acquisition day 5 in male rats exposed to BMAA on PND 3 (Figure 6A,B). A similar trend can be seen in that of female BMAA-exposed (Figure 7A,B). PND 4 and 5 exposed male and female rats showed marginal but insignificant improvement relevant to the control, and PND 6 and 7 showed a substantial, but not significant, improvement relative to the PND 3 rats. Performance of male rats exposed to BMAA on PND 7 improved considerably more than that of female rats exposed on the same day, which could suggest that females are more susceptible to BMAA toxicity than males on PND 7 or, alternatively, that the estrous cycle of this group could have negatively influenced this result. The overall lack or low percentage of improvement observed in male and female BMAA-exposed rats, indicates that rats exposed to BMAA on PND 3, 4 and 5 had trouble with consolidating learned information about a maze into their reference memory. 

Long-term reference memory testing, evaluated using the radial arm retention test one week after acquisition trails ended, demonstrated that control and vehicle control rats were able to retain the learned pattern of baited arms, whereas neither male (Figure 6C) nor female (Figure 7C) rats exposed to BMAA on especially PND 3, 4 and 5 were unable to recall what they had learned in the acquisition trials. This suggests that BMAA, when administered at these ages, greatly impairs the long-term memory of both male and female rats. Karlsson et al. [19] observed some short-term reference memory impairment in rats exposed to 650 mg/kg BMAA on PND 9 and PND 10, but the retention test revealed no differences between the treated and control groups in their study. The lack of long term memory impairment following exposure to 650 mg/kg BMAA on PND 9 and 10 compared to the severe memory impairment seen in rats exposed to only 400 mg/kg BMAA but on PND 3, 4 or 5 highlights the importance of age of exposure over exposure dose.

**Figure 8:**
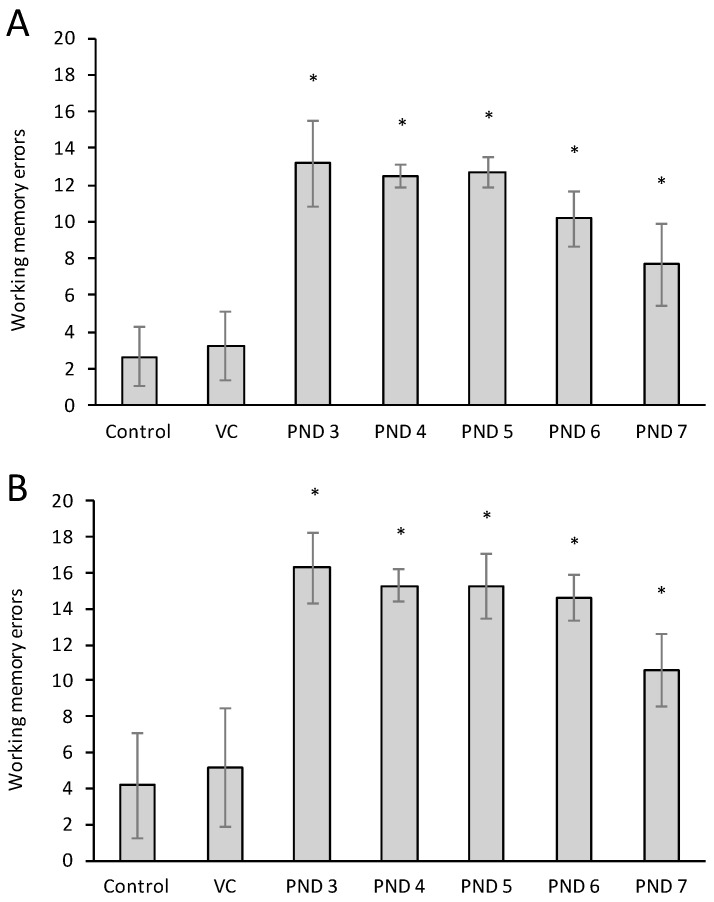
Working memory errors observed in male (**A**) and female (**B**) Sprague Dawley rats neonatally exposed to 400 mg/kg BMAA compared to control and vehicle control rats. Errors across all acquisition trials were pooled (*n* = 75 for vehicle control and *n* = 25 for all other groups). (*) Indicate significant difference from the control (*p* < 0.05).

**Figure 9:**
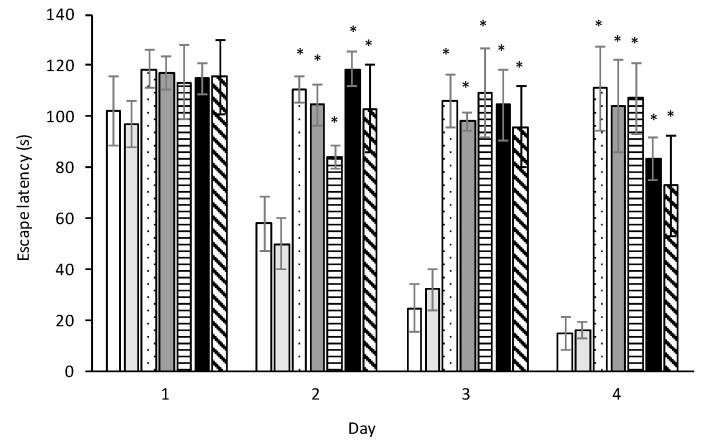
Recorded escape latencies, time to the platform, tested in the Morris water maze hidden platform test for control (white bar) and vehicle control rats (light grey bar) compared to rats exposed to 400 mg/kg BMAA on PND 3 (dotted bar), PND 4 (dark grey bar), PND 5 (horizontally striped bar), PND 6 (black bar) and PND 7 (diagonally striped bar) (*n* = 30 for vehicle control and *n* = 10 for all other groups). Normal, healthy rats learn to use spatial cues to navigate itself towards the hidden platform; they memorize this location and recall it in subsequent trials, and has progressively shorter latencies throughout the study (as seen for the control and vehicle control rats). (*) Indicates significant difference from the control (*p* < 0.05).

Working memory is responsible for the temporary storing and managing of information required to carry out immediate complex cognitive tasks such as the rat remembering which arm had already been visited during that specific trial. Working memory errors were significantly higher in male rats exposed to BMAA from PND 3 up to PND 7 (Figure 8A) and in female rats exposed at the same ages (Figure 9B), with male rats more severely impaired. Working memory impairment is a well-recognized characteristic of AD [87]. 

The performance in the radial arm maze is predominantly influenced by the hippocampus [88], mainly because the hippocampus is responsible for continually updating the position of the animal during its navigation to a goal [89] and due to its involvement in reference and working memory retention. Thus, the inability of BMAA-exposed rats to perform in this paradigm suggests that the hippocampus could be a potential target of BMAA toxicity. Interestingly, Buenz and Howe [90] also reported that the hippocampus is the neuronal population that is the most sensitive to BMAA toxicity, while Karlsson et al. [20] demonstrated that there is distinct uptake and selective localization of BMAA in the hippocampus of the neonatal rodent brain and not in that of the adult brain, which could contribute to the high level of BMAA toxicity observed in neonatally exposed rats in this study compared to in previous BMAA toxicity evaluations that were conducted on adult rats. Furthermore, the reduced latency in first pellet acquisition as a function of learning, was less marked in PND 3, 4 and 5 than in the control groups. This change in performance was not due to a decreased food motivation in BMAA-exposed rats, as there was no difference in average body weight between the control rats and treated rats at any time during the test, and all rats consumed pellets from baited arms immediately after collection. Additionally, the same memory deficits were observed in exposed rats upon testing in the Morris water maze (discussed below), which is not a food-motivated task.

### 2.8. Ihe Morris Water Maze

Escape latency reflects a rat’s ability to navigate itself in the water maze, using the spatial cues provided and its memory of the previous location of the platform, towards a hidden platform in the water bath. Young, unimpaired rats can learn and memorize the position of the platform within a few days, and subsequently accomplish this task in less than 20 s [91], as shown for the control and vehicle control rats of this study. Figure 9 shows the negative effect of BMAA on short-term memory and learning, with escape latencies remaining more or less constant for rats exposed to BMAA on PND 3, 4 and 5. Rats exposed on PND 6 and 7 shows significant improvement in this task over the four days testing period compared to the PND 3 rats, but is still significantly different from the control. 

**Figure 10:**
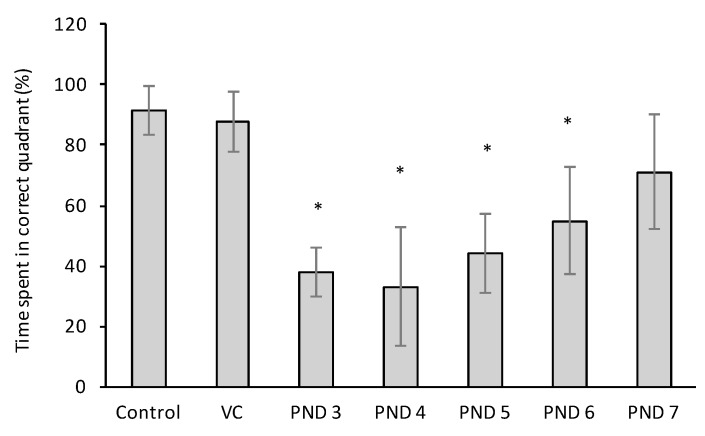
Percentage time spent in the correct quadrant, where the platform was located in the previous trials, in the probe trial (where platform is removed) of the Morris water maze observed in control, vehicle control and BMAA-treated Sprague Dawley rats (*n* = 30 for vehicle control and *n* = 10 for all other groups) as tested three days after the acquisition trials. (*) Indicates significant difference from the control (*p* < 0.05).

The time spent in the correct quadrant of the water maze after removal of the sub-surface platform, the probe trial, is an indication of the long-term memory ability of the rats [92]. BMAA exposure had a significant and relatively large negative effect on this for PND 3, 4, and 5 exposed rats, a significant but lesser effect on PND 6 exposed rats, and no significant effect on PND 7 exposed rats (Figure 10). From this clear variance in performance observed between rats exposed to BMAA at different neonatal ages it is evident that age of exposure is a major determinant of BMAA toxicity and that exposure to BMAA on PND 3 is substantially more damaging than exposure to BMAA at a later age. The Morris water maze is considered a valid method for investigating effects on hippocampal dependent spatial navigation and reference memory, and Sutherland and Hoesing [93] specifically indicated that rats that do poorly in post-training probe trials generally have significant neuronal loss or lesions in the hippocampus and dentate gyrus.

Whether observed abnormalities or inability to perform in cognition testing are true reflections of memory impairment, or whether they are rather a function of slight locomotor impairment, is often debated. However, unlike the radial arm maze, Voorhees and Williams [94] showed that performance on the Morris water maze is independent of locomotor effects since land-based locomotor deficit does not affect swimming speed. Furthermore, it is accepted that when the experimental subjects have deficits during the probe trials specifically, learning and/or memory is further dissociated from locomotor performance because measures recorded on probe trials are unaffected by swimming speed [94]. Thus, impairments shown in the radial arm maze and confirmed using the Morris water maze in this study are completely locomotor activity independent, and unequivocally show that BMAA exposure on PND 3, but also on PND 4, 5 and 6, causes short and long-term memory deficits in rats. 

## 3. Conclusions

Although it has previously been suggested that BMAA might be a developmental toxin [17,19,20,28,31,32] these suggestions lacked any supporting behavioral and/or cognitive assessment or neuropathology, and the observed symptoms that were reported did not correlate well to those typically observed in AD and/or PD patients. This is, to our knowledge, the first study where true AD and PD-like symptoms could simultaneously be observed in rats following BMAA exposure. This is also the first study that demonstrates the importance of age of exposure over total BMAA exposure dose, with 400 mg/kg at PND 3 being significantly more toxic than 650 mg/kg at PND 9 and 10 [19,20] and a single exposure at PND 5 being just as toxic as an accumulative exposure dose administered *in utero*, on PND 5 and on PND 10. 

The data presented here show that when neonatal rats were exposed to BMAA on PND 3, 4 and 5 several AD and/or PD-related behavioral and cognitive deficits could be observed simultaneously. Male rats exhibited severe unilateral hind limb splay while whole body tremors could be observed in exposed female rats. Eye opening, a developmental landmark in rats, was delayed by two days in male and female rats exposed to BMAA on PND 3, 4 or 5. Furthermore, male and female BMAA-exposed rats failed to identify and discriminate a learned odor, an early non-motor symptom of PD, and exhibited decreased locomotor activity, decreased exploration and increased anxiety in the open field test. Alterations were also observed in the rats’ natural passive defense mechanism, which is typically associated with interference with the developing serotonergic and dopaminergic system. Potential memory deficits, and changes to the rat’s natural height avoidance behavior, could be observed as early as PND 30 when tested in the elevated plus maze and were confirmed using the radial arm maze and Morris water maze. Spatial learning, short term working and reference memory and long-term memory were impaired in male and female rats exposed to BMAA on PND 3, 4, 5, 6 and 7. As several of the BMAA-induced symptoms reported here are typically associated with the dysfunction of the hippocampus and/or dentate gyrus it is tempting to speculate on the possibility that, in rodents, BMAA is selectively toxic to the hippocampal neuronal population which would corroborate the findings of Buenz and Howe [90]. Additionally, several of the toxic effects seen here in adult rats following neonatal BMAA exposure are indicative of dysfunctional dopamine and/or serotonin signaling. Similarly, the clinical symptoms observed following BMAA exposure on especially PND 3, 4 and 5 are similar to those observed in rodents following exposure to MDMA, a monoamine reuptake inhibitor and VMAT inhibitor, and to reserpine, also a VMAT inhibitor, which further supports the notion that BMAA could potentially interfere with dopamine and/or serotonin systems. Interestingly, alterations in the levels of dopamine and/or serotonin as a result of BMAA exposure have been reported in rodents. Santiago et al. [95] observed a dose-dependent increase in the extracellular output of dopamine in the striatum of adult rats following perfusion of BMAA into this brain region. Dawson et al. [31] furthermore reported alterations in the CSF dopamine and serotonin levels in neonatally exposed BMAA-rats compared to control rats. Although Lindstrom et al. [96] did not observe any alterations in dopamine and serotonin levels in several brain structures following an intracisternal BMAA injection, they did observe dopaminergic neuronal loss and pyknotic neurons in the substantia nigra surrounding the BMAA injection site (and not following an injection with the vehicle control). The observed neuronal loss in the substantia nigra can in itself cause alterations in dopaminergic transmission. These data suggest that BMAA could, in fact, alter dopaminergic and/or serotonergic signaling in exposed rats. Herlenius and Lagercrantz [97] demonstrated that the disruption of the normal timing or intensity of neurotransmitter signaling during the critical phases of brain development can lead to permanent changes in proliferation, differentiation and growth of their target cells and suggested this to provide the underlying mechanism for neurobehavioral abnormalities in adulthood, and this could well be a means by which BMAA elicits its toxicity. Since rat pups are born precociously compared to humans, with brain development in a PND1-PND4 rat comparable to a pre-term infant (second trimester of pregnancy), that of a PND 7–9 rat to the brain of an infant at approximately 36 weeks of gestation and that of a PND 10 rat to an infant at birth [38,98] the relevance of postnatal rat exposure to human exposure should be carefully considered. A full histopathological examination of the brain and spinal cord tissue of the rats used in this study will give more insight into the brain structures and/or target areas affected by neonatal BMAA exposure. 

## 4. Materials and Methods

### 4.1. Chemicals

β-*N*-methylamino-l-alanine (BMAA) was purchased from The Brain Chemistry Labs, Wyoming, USA and the purity confirmed as described by Banack et al. [99].

### 4.2. Animal Maintenance

Pregnant Sprague Dawley rats were obtained from North-West University Animal Facility (South Africa) at gestational (G) day 12. The dams were housed alone in polypropylene cages with wired mesh lids containing wood shaving bedding, nesting material and enrichment items. The litters were cross-fostered at PND 3. Pups were divided into exposure groups by random selection. Each litter contained a minimum of 10 pups, with a homogeneous distribution of males and females as far as possible. The litter weights were monitored on PND 1, 4, 7, 9, 14, 19, and 22 and after that every two weeks. After weaning on PND 23 and onwards, five gender-matched rats were housed together in their respective treatments groups in standard polypropylene cages containing wood shaving bedding and enrichment items. The animals were maintained on a standard rodent diet (Epol^®^ rodent pellets) that was, together with water, provided *ad libitum*, unless otherwise stated, and housed in a temperature and humidity controlled environment with a 12 h light cycle beginning at 05:30. Animals were randomized into exposure groups and identified by numerical markings on the tails. All animal experiments were approved by the Nelson Mandela Metropolitan University Research Ethics Committee—Animal (Project reference number A15-SCI-BCM-001; Approved on 23 May 2015) and were conducted in accordance with national and institutional guidelines for the protection of animal welfare. 

### 4.3. Exposure

L-BMAA hydrochloride was dissolved in standard Hanks Balanced Salt Solution (HBSS) and filtered using Corning^®^ Costar Spin-x centrifuge filter tubes, and a single dose of 400 mg/kg BMAA injected subcutaneously in a volume of 5 mL/kg body weight into both male and female Sprague Dawley pups on postnatal day 3, 4, 5, 6, 7 or 10. Vehicle control animals received equivalent injections of HBSS vehicle, which does not itself produce any toxicity. Three HBSS vehicle control groups were used for each gender. One group was exposed to a cumulative vehicle control dose (on G14, PND 5 and PND 10), a second group exposed to vehicle control on PND 3 only, and a third group exposed to vehicle control on PND 5 only. There were no differences between any of these vehicle control groups (and also no differences between any vehicle control group and the control group) and the data from of vehicle controls were pooled. A 31-gauge, 8mm needle attached to a 0.5-mL insulin syringe (BD Ultra-Fine^®^) was used to inject BMAA and/or vehicle into neonatal rats. Pregnant dams were injected with the same dose (per weight of dam) on gestation day 14 to evaluate pre-natal exposure. Another exposure group was exposed to BMAA prenatally and again on postnatal days 5 and 10 to test the effect of an accumulative BMAA dose. 

### 4.4. Behavioural Analysis

**Figure 11:**
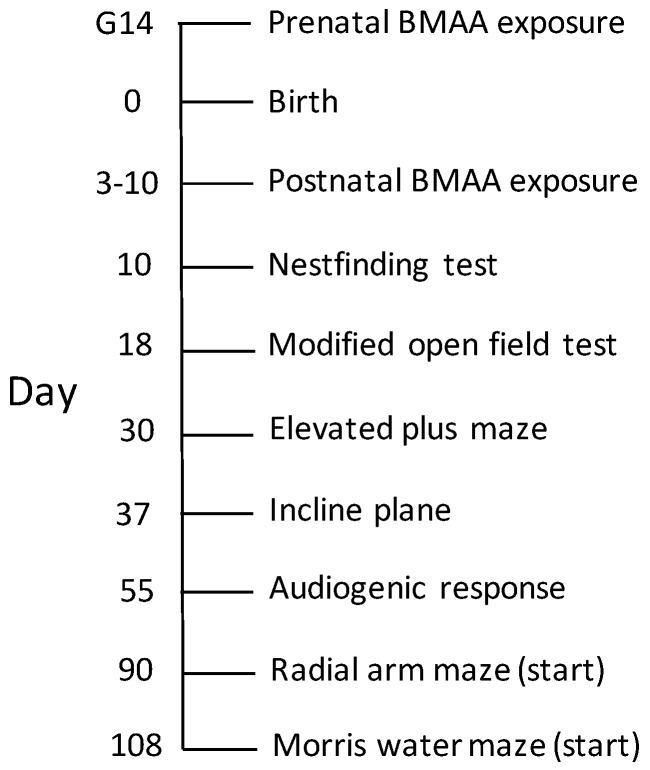
Experimental design timeline indicating ages of BMAA exposure together with the days behavioral, emotional response and cognitive tests listed below were conducted.

All animals were subjected to standard behavioral testing as per Figure 11. So as to avoid any increase in general activity and stress in animals, behavioral tests were never conducted on the same day that the rodents’ home cages were scheduled for changing. Test room lighting, temperature, and noise levels were also kept consistent for all test subjects. The same female experimenter conducted all behavioral and cognitive tests for all control and exposure groups. Prior to testing, all relevant behavioral equipment was wiped down with 70% ethanol. Additionally, after each subject completed its test session, fecal boli and urine were removed, surfaces wiped thoroughly with 70% ethanol and a deodorizer (F10^®^ veterinary disinfectant and odor eliminator), and the test chamber allowed to dry completely before starting another subject. 

#### 4.4.1. The Nestfinding Test on Postnatal Day 10

The standard nest finding test, first described by Meyerson [64], allows for the early observation of locomotor dysfunction and/or evaluates olfactory abnormalities. An arena of 42 × 26 × 18 cm was divided into two equal areas with a nest at each end. One of the nests consisted of bedding material from the home cage and the other nest was made of fresh bedding material. Each pup was placed individually in the center of the arena, starting in an orientation perpendicular to its nest position, and allowed to find his or her home nest over a period of 90 s. At PND 10 the rat is solely dependent on smell to locate its own nest, which, at this age, it will preferentially try to reach [64]. The test was repeated three times for each pup, each time with a starting point 180° from the previous time. Time to reach the nest was recorded. If the nest was not reached, the time was recorded as 90 s (maximum time allowed). 

#### 4.4.2. The Modified Open Field Test on Postnatal Day 20

The modified open field test allows the evaluation of general locomotor ability, overall activity, and novel environment exploration, and provides an initial screening for anxiety-related behavior in young rats, and was conducted as described by Lamprea et al. [100], Karlsson et al. [20] and Bailey and Crawley [101]. Rats were placed in the middle of an arena, 42 × 26 × 18 cm, and allowed to explore the novel environment for 10 min. Black lines drawn on the floor of the area divided the floor into sixteen rectangles. The central square of the arena was marked with a red cross. All animals were tested once. The following variables were quantified:

Centre square entries: Frequency with which the rat entered the central X-marked area with all four paws.

Line crossing: Frequency with which rat crossed a line with all four paws. 

Stationary time: Time rat was completely stationary.

Rearing: Frequency with which rats stood on their hind legs in the arena.

Urination: Number of puddles or streaks of urine.

Defecation: Number of fecal boli produced. 

Unusual posture or behavior, such as hind leg splay, circling, tail dragging and body tremors, were also recorded for each rat. 

Number of lines crossed indicates general locomotor activity while total time stationary is similarly a function of locomotor inactivity. The number of central square entries and the duration of time spent in the central square indirectly measure thigmotaxis, which is an indicator of exploratory behavior and anxiety in rats. A high frequency/duration of central square entries indicates high exploratory behavior and low anxiety levels, whereas anxiousness will result in a rat remaining close to the walls of the arena throughout the test. Rearing is considered an indicator of exploratory behavior [102]. 

#### 4.4.3. The Elevated Plus Maze Test on Postnatal Day 30

The elevated plus maze is used as an assay of anxiety-related behavior in rats. Tests were conducted exactly as described by Walf and Frye [83] and Karlsson et al. [19,20]. The time spent on each arm and entries made on the open and closed arms were recorded. Other ethological parameters such as rearing, freezing, stretch-attend postures and head dips were also recorded. Activity in the open arms reflects a conflict between the rodent’s proclivity for enclosed spaces (closed arms) and their innate motivation to explore novel environments [83]. An increase in the duration of open arm activity reflects anti-anxiety behavior, whereas repeated entries into the same arm could indicate memory impairment or an alteration in the rat’s natural behavior. All animals were tested once. The maze was cleaned with 70% ethanol and an odor eliminating spray after each animal. 

#### 4.4.4. The Inclined Plane Test on Postnatal Day 37

The inclined plane test, as described by Roos et al. [103] was used to test muscle performance of rats. The apparatus consisted of a rectangular box (50 × 25 × 18 cm) with a rubber-coated floor. One end of the box could be lifted off the base, fitted with a protractor, to establish an inclined plane. The maximum angle at which the rats could maintain themselves, before sliding down the floor, was recorded. Each rat was tested three times and the average angle designated as its score. 

#### 4.4.5. The Audiogenic Freezing Response on Postnatal Day 55

The audiogenic immobility, or freezing reaction test, evaluates a prominent component of the rat’s passive defensive mechanism that attains adult level around 40 days of age. In response to a sudden noise, the animal stops any ongoing activity and freezes. This task was conducted as described by Hard et al. [85] and Karlsson et al. [20]. Rats were allowed to adapt to the environment, an arena of 42 × 26 × 18 cm, for 5 min before the freezing response was triggered by a 5 s noise (120 dB). The duration of immobility (defined as the time from the freezing response to the first distinct movement of some part of the body) was recorded for each rat. Observations were terminated if rat remained immobile for more than 10 min and the time immobile was recorded as 10 min. All animals were tested once. The area was cleaned with 70% ethanol between each rat.

#### 4.4.6. The Radial Arm Maze Test from Postnatal Day 90

The radial arm maze is a paradigm that commonly assesses spatial learning, working and reference memory in rodents [104,105,106]. The apparatus used was an eight-arm radial maze in mild steel elevated 45 cm above the floor. Each arm (50 cm long and 10 cm wide) has low side-walls (15 cm), which prevent the rats from jumping from one arm to another and extends radially from a central platform that is 32 cm in diameter. The ends of the arms are not closed, which allows use of extra-maze visual cues from within the maze and specifically from the food cups. Rats began training in the maze at 90 days of age. During the training and trials rats had *ad libitum* access to water in their home cages, but with daily feeding given after testing, at sufficient amounts to maintain rats at a healthy body weight of 85% that of the initial free-feeding weight. Rats were habituated to the maze for 10 min daily for two consecutive days in their experimental groups (*n* = 5/sex/treatment), allowing the rats to familiarize themselves with the maze and spatial cues, before the acquisition trials started. In the acquisition trials, where each rat was tested once daily for five consecutive days, four out of the eight arms were baited with a small food pellet (Supreme petfood^®^ rat treats were used to increase rats’ motivation to collect food pellets) placed in a cup 0.5 cm from the distal end of each arm, while the other four arms were always left unbaited to test reference memory in subsequent sessions. The pattern of baited and unbaited arms was consistent throughout testing for each rat but differed for each rat in the treatment group. Each trial began by placing the rat on the central platform, from where the rat could not see which food cups were baited, and then allowed to freely enter any arm. The test session lasted for 10 min, or until all eight arms were entered. Arms were baited only once per session, thus any food collected from the cups was not replaced in the 10-min session, and a repeated entry into a baited arm during the same session was counted as a working memory error, whereas entrance into an unbaited arm was recorded as a reference memory error. The test thus involved two parameters of memory function: (a) working memory error, re-entries into arms from which the reinforcer had already been retrieved within a trial, and (b) reference memory which is entry into arms that have never been baited. A decrease in the reference memory over the five days testing period is known as the learning curve; a steeper curve represents faster task learning [106]. The same test was repeated once one week after the last acquisition session to assess the rat’s memory retention.

#### 4.4.7. Morris Water Maze Test from Postnatal Day 108

The Morris water maze is a behavioral task that specifically evaluates hippocampal-dependent learning and memory by requiring an animal to locate a hidden platform, submerged below the water surface, after learning the platform’s fixed location. The test arena was set up as described by Wenk [105] and Bromley-Brits et al. [92]. Each rat was gently placed into the water, facing the edge of the pool and started from positions distal from the platform (three trials per rat per day, each from a different starting position). Rats were given 120 s to locate the platform in the pool where it was allowed to stay on the platform for 5 s before being dried and returned to the home cage. If a rat were unable to find the platform within the designated 120 s, the rat was guided towards the platform and allowed to stay on the platform for 20 s before being dried and returned to the home cage. For the following four consecutive test days, Days 1–4, the platform was submerged 1 cm below the water surface (but at the same position as before), and thus invisible to the rat, and the rats were required to locate the invisible platform by remembering it is previous position in the pool. The escape latency was recorded for each rat. A change in escape latency over the four days of the hidden platform test is known as the learning curve; a steeper curve represents faster task learning (Nunez, 2008). Finally, a probe trial was done on day 7, three days after the last acquisition trial, where the platform was removed from the pool. The time spent in the quadrant that previously contained the platform was recorded; a higher percentage of time spent in this quadrant can be interpreted as a higher level of memory retention [92].

### 4.5. Behavioural Recordings

The animals were monitored by video recordings of all behavioral tests. All behavioral tests were recorded on video, blinded and each video watched three times to time and/or record specific events as listed above before un-blinding. 

### 4.6. Statistical Analysis

Due to the number of replicates in the study, no assumptions concerning equal sample variance or normal distribution of data were made, and the non-parametric Mann-Whitney *U* test (α = 0.05) was used to determine the statistical validity of data presented here.

## 5. Patents

Downing, T.G. (2017) Animal Model of Neurodegenerative Disease. United Kingdom—Provisional Patent Application No. 1705535.1 in the name of Nelson Mandela Metropolitan University entitled; Filing date: 5 April 2017.

## Figures and Tables

**Table 1:** Percentage of rats that were able to find their nest in the allocated time in each trial of the nest finding test (*n* = 30 for vehicle control and *n* = 10 for all other groups).

Treatment Group	Trial 1	Trial 2	Trial 3
Control	100%	100%	100%
Vehicle control	100%	100%	100%
G14	100%	80%	90%
PND 3	60%	60%	60%
PND 4	60%	70%	70%
PND 5	60%	60%	80%
PND 6	80%	80%	80%
PND 7	100%	100%	80%
PND 10	90%	90%	100%

## References

[1] Dietrich D.R., Fischer A., Michel C., Hoeger S.J. (2008). Toxin mixture in cyanobacterial bloom—A critical comparison of reality with current procedures employed in human health risk assessment. Cyanobact. Harmful Algal Blooms.

[2] Brand L.E. (2009). Human exposure to cyanobacteria and BMAA. Amyotroph. Lateral Scler..

[3] Brand L.E., Pablo J., Compton A., Hammerschlag N., Mash D.C. (2010). Cyanobacterial blooms and the occurrence of the neurotoxin, β-*N*-methylamino-l-alanine (BMAA), in South Florida aquatic food webs. Harmful Algae.

[4] Jonasson S., Eriksson J., Berntzon L., Spáčil Z., Ilag L.L., Ronnevi L.-O., Rasmussen U., Bergman B. (2010). Transfer of a cyanobacterial neurotoxin within a temperate aquatic ecosystem suggests pathways for human exposure. Proc. Natl. Acad. Sci. USA.

[5] Mondo L., Hammerschlag N., Basile M., Pablo J., Banack S.A., Mash D.C. (2012). Cyanobacterial neurotoxin β-*N*-methylamino-l-alanine (BMAA) in shark fins. Mar. Drugs.

[6] Downing S., Contardo-Jara V., Pflugmacher S., Downing T.G. (2014). The fate of the cyanobacterial toxin β-*N*-methylamino-l-alanine in freshwater mussels. Ecotoxicol. Environ. Saf..

[7] Contardo-Jara V., Schwanemann T., Pflugmacher S. (2014). Uptake of a cyanotoxin β-*N*-methylamino-l-alanine (BMAA), by wheat (*Triticum aestivum*). Ecotoxicol. Environ. Saf..

[8] Al-Sammak M.A., Hoagland K.D., Cassada D., Snow D.D. (2014). Co-occurrence of the cyanotoxins BMAA, DABA and Anatoxin-A in Nebraska Reservoirs, Fish and Aquatic Plants. Toxins.

[9] Mondo K., Glover B.W., Murch S.J., Liu G., Cai Y., Davis D.A., Mash D.C. (2014). Environmental neurotoxins β-*N*-methylamino-l-alanine (BMAA) and mercury in shark cartilage dietary supplements. Food Chem. Toxicol..

[10] Jiang L., Kiselova N., Rosen J., Ilag L.L. (2014). Quantification of neurotoxin BMAA (β-*N*-methylamino-l-alanine) in seafood from Swedish markets. Sci. Rep..

[11] Lage S., Annadotter H., Rasmussen U., Rydberg S. (2015). Biotransfer of β-*N*-methylamino-l-alanine (BMAA) in a Eutrophicated Freshwater Lake. Mar. Drugs.

[12] Reveillon D., Abadie E., Sechet V., Masseret E., Hess P., Amzil Z. (2015). β-*N*-methylamino-l-alanine (BMAA) and isomers: Distribution on different food web compartments of Thau lagoon, French Mediterranean Sea. Mar. Environ. Res..

[13] Reveillon D., Sechet V., Hess P., Amzil Z. (2016). Systemic detection of BMAA (β-*N*-methylamino-l-alanine) and DAB (2,4-diaminobutyric acid) in mollusks collected in shellfish production areas along the French coasts. Toxicon.

[14] Cox P.A., Sacks O.W. (2002). Cycad neurotoxins, consumption of flying foxes, and ALS-PDC disease in Guam. Neurol.

[15] Murch S.J., Cox P.A., Banack S.A. (2004). A mechanism for slow release of biomagnified cyanobacterial toxins and neurodegenerative disease in Guam. Proc. Natl. Acad. Sci. USA.

[16] Seawright A.A., Brown A.W., Nolan C.C., Cavanagh J.B. (1990). Selective degeneration of cerebellar cortical neurons caused by cycad neurotoxin l-b-methylaminoalanine (BMAA), in rats. Neuropathol. Appl. Neurobiol..

[17] Vega A., Bell E.A., Nunn P.B. (1968). The preparation of l- and d-α-amino-bmethylaminopropionicacids and the identification of the compound isolated from Cycas circinalis as the l-isomer. Phytochemistry.

[18] Polsky F.I., Nunn P.B., Bell E.A. (1972). Distribution and toxicity of amino-b-methylaminopropionic acid. Fed. Proc..

[19] Karlsson O., Roman E., Brittebo E.B. (2009). Long-term cognitive impairments in adult rats treated neonatally with beta-*N*-Methylamino-l-Alanine. Toxicol. Sci..

[20] Karlsson O., Lindquist N.G., Brittebo E.B., Roman E. (2009). Selective Brain Uptake and Behavioural Effects of the Cyanobacterial Toxin BMAA (b-*N*-Methylamino-l-alanine) following Neonatal Administration to Rodents. Toxicol. Sci..

[21] Al-Sammak M.A., Rogers D.G., Hoagland K.D. (2015). Acute B-*N*-Methylamino-l-alanine Toxicity in a mouse model. J. Toxicol..

[22] Rakonczay Z., Matsuoka Y., Giacobini E. (1991). Effects of l-β-*N*-methylamino-l-alanine (l-BMAA) on the cortical cholinergic and glutamatergic systems of the rat. J. Neurosci. Res..

[23] Chang Y.-C., Chiu S.-J., Kao K.-P. (1993). Beta-*N*-methylamino-l-alanine (l-BMAA) decreases brain glutamate receptor number and induces behavioral changes in rats. Chin. J. Physiol..

[24] Perry T.L., Bergeron C., Biro A.J., Hansen S. (1989). Chronic oral administration of b-*N*-methylamino-l-alanine is not neurotoxic to mice. J. Neurol. Sci..

[25] Duncan M.W., Villacreses N.E., Pearson P.G., Wyatt L., Rapoport S.I., Kopin I.J., Markey S.P., Smith Q.R. (1991). 2-Amino-3-(methylamino)-propanoic acid (BMAA) pharmacokinetics and blood–brain barrier permeability in the rat. J. Pharmacol. Exp. Ther..

[26] Cruz-Aguado R., Winkler D., Shaw C.A. (2006). Lack of behavioral and neuropathological effects of dietary β-methylamino-l-alanine (BMAA) in mice. Pharmacol. Biochem. Behav..

[27] Scott L.L., Downing S., Downing T.G. (2017). The evaluation of BMAA inhalation as a potential exposure route using a rat model. Neurotox. Res..

[28] Smith Q.R., Nagura H., Takada Y., Duncan M.W. (1992). Facilitated transport of the neurotoxin, beta-*N*-methylamino-l-alanine, across the blood–brain barrier. J. Neurochem..

[29] Banos G., Daniel P.M., Pratt O.E. (1978). The effect of age upon the entry of some amino acids into the brain, and their incorporation into cerebral protein. Dev. Med. Child Neurol..

[30] O’Tuama L.A., Phillips P.C., Smith Q.R., Uno Y., Dannals R.F., Wilson A.A., Ravert H.T., Loats S., Loats H.A., Wagner H.N. (1991). l-methionine uptake by human cerebral cortex: Maturation from infancy to old age. J. Nucl. Med..

[31] Dawson R., Marschall E.G., Chan K.C., Millard W.J., Eppler B., Patterson T.A. (1998). Neurochemical and neurobehavioral effects of neonatal administration of b-methylamino-l-alanine and 3,3′iminodipropionitrile. Neurotoxicol. Teratol..

[32] Karlsson O., Roman E., Berg A.L., Brittebo E.B. (2011). Early hippocampal cell death, and late learning and memory deficits in rats exposed to the environmental toxin BMAA (β-*N*-methylamino-l-alanine) during the neonatal period. Behav. Brain Res..

[33] Karlsson O., Berg A.-L., Lindstrom A.-K., Arnerup G., Roman E., Bergquist J., Hanrieder J., Lindquist N.G., Brittebo E.B., Andersson M. (2012). Neonatal exposure to the cyanobacterial toxin BMAA induces changes in protein expression, and neurodegeneration in adult hippocampus. Toxicol. Sci..

[34] Dobbing J., Sands J. (1979). Comparative aspects of the brain growth spurt. Early Hum. Dev..

[35] Gottlieb A., Keydar I., Epstein H.T. (1977). Rodent brain growth spurts: An analytical review. Biol. Neonate.

[36] Baloch S., Verma R., Huang H., Khurd P., Clark S. (2009). Quantification of brain maturation and growth patterns in C57Bl/6 mice via computational neuroanatomy of diffusion tensor images. Cereb. Cortex.

[37] Bockhorst K.H., Narayana P.A., Liu R., Ahobila-Vijjula P., Ramu J. (2008). Early postnatal development of rat brain: In vivo diffusion tensor imaging. J. Neurosci. Res..

[38] Semple B.D., Blomgren K., Gimlin K., Ferriero D.M., Noble-Haeusslein L. (2013). Brain development in rodents and humans: Identifying benchmarks of maturation and vulnerability to injury across species. Prog. Neurobiol..

[39] Garruto R.M., Gajdusek D.C., Chen K.M. (1981). Amyotrophic lateral sclerosis and Parkinsonism dementia among Filipino migrants to Guam. Ann. Neurol..

[40] Sabel C.E., Boyle P.J., Löytönen M., Gatrell A.C., Jokelainen M., Flowerdew R., Maasilta P. (2003). Spatial clustering of amyotrophic lateral sclerosis in Finland at place of birth and place of death. Am. J. Epidemiol..

[41] Carvalho M.M., Campos F.L., Coimbra B., Pêgo J.M., Rodrigues C., Lima R., Rodrigues A.J., Sousa N., Salgado A.J. (2013). Behavioural characterization of the 6-hydroxidopamine model of Parkinson’s disease and pharmacological rescuing of non-motor deficits. Mol. Neurodegener..

[42] Meredith G.E., Kang U.J. (2006). Behavioral models of Parkinson’s disease in rodents: A new look at an old problem. Mov. Disord..

[43] Betarbet R., Sherer T.B., MacKenzie G., Garcia-Osuna M., Panov A.V., Greenamyre J.T. (2000). Chronic systemic pesticide exposure reproduces features of Parkinson’s disease. Nat. Neurosci..

[44] Schmidt W.J., Alam M. (2006). Controversies on new animal models of Parkinson’s disease pro and con: The rotenone model of Parkinson’s disease (PD). J. Neural Transm. Suppl..

[45] Tole S., Christian C., Grove E.A. (1997). Early specification and autonomous development of cortical fields in the mouse hippocampus. Development.

[46] Lee S.M., Tole S., Grove E., McMahon A.P. (2000). A local Wnt-3a signal is required for development of the mammalian hippocampus. Development.

[47] Tole S., Grove E.A. (2001). Detailed field pattern is intrinsic to the embryonic mouse hippocampus early in neurogenesis. J. Neurosci..

[48] Altman J., Bayer S.A. (1995). Atlas of Prenatal Rat Brain Development.

[49] Huang H., Liu C.M., Sun J., Hao T., Xu C.M., Wang D., Wu Y.Q. (2016). Ketamine Affects the Neurogenesis of the Hippocampal Dentate Gyrus in 7-Day-Old Rats. Neurotox. Res..

[50] Voorn P., Kalsbeck A., Jorritsma-Byham B., Groenewegen H.J. (1988). The pre- and postnatal development of the dopaminergic cell groups in the ventral mesencephalon and the dopaminergic innervation of the stria- tum of the rat. Neuroscience.

[51] Tepper J.M., Damlama M., Trent F. (1994). Postnatal changes in the dis- tribution and morphology of rat substantia Nigra dopaminergic neurons. Neuroscience.

[52] Schmidt U., Beyer C., Oestreicher A.B., Reisert I., Schilling K., Pilgrim C. (1996). Activation of dopaminergic D1 receptors promotes morphogenesis of developing striatal neurons. Neuroscience.

[53] Spencer G.E., Klumperman J., Syed N.I. (1998). Neurotransmitters and neurodevelopment. Role of dopamine in neurite outgrowth, target selection and specific synapse formation. Perspect. Dev. Neurobiol..

[54] Stanwood G., Levitt P., Nelson C.A., Luciana M. (2001). The effects of cocaine on the developing nervous system. Handbook of Developmental Cognitive Neuroscience.

[55] Bellone C., Mameli M., Luscher C. (2011). In Utero exposure to cocaine delays postnatal synaptic maturation of glutamatergic transmission in the VTA. Nat. Neurosci..

[56] McCarthy D.M., Zhang X., Darnell S.B., Sangrey G.R., Yanagawa Y., Sadri-Vakili G. (2011). Cocaine alters BDNF expression and neuronal migration in the embryonic mouse forebrain. J. Neurosci..

[57] Goldstein J.M., Barnett A., Malick J.B. (1975). The evaluation of anti-Parkinson drugs on reserpine-induced rigidity in rats. Eur. J. Pharmacol..

[58] Green A.R., Backus L.I. (1990). Animal models of serotonin behavior. Ann. N. Y. Acad. Sci..

[59] Fitzgerald J.L., Reid J.J. (1990). Effects of methylenedioxymethamphetamine on the release of monoamines from rat brain slices. Eur. J. Pharmacol..

[60] Eiden L.E., Weihe E. (2011). VMAT2: A dynamic regulator of brain monoaminergic neuronal function interacting with drugs of abuse. Ann. N. Y. Acad. Sci..

[61] Hamers F.P.T., Lankhorst A.J., Van Laar T.J., Veldhuis W.B., Gispen W.H. (2001). Automatedquantitative gait analysis during overground locomotion inthe rat: Its application to spinal cord contusion and transec-tion injuries. J. Neurotrauma.

[62] Bakke J.L., Lawrence N.L., Robinson S.A., Bennett J., Bowers C. (1978). Late endocrine effects of l-dopa, 5-HTP, and 6-OH-dopa administered to neonatal rats. Neuroendocrinology.

[63] Glazova N.Y., Merchieva S.A., Volodina M.A., Sebentsova E.A., Manchenko D.M., Kudrun V.S., Levitskaya N.G. (2014). Effects of Neonatal Fluvoxamine Administration on the Physical Development and Activity of the Serotoninergic System in White Rats. Acta Nat..

[64] Meyerson B.J. (1985). Influence of early beta-endorphin treatment on the behavior and reaction to beta-endorphin in the adult male rat. Psychoneuroendocrinology.

[65] Kranick S.M., Duda J.E. (2008). Olfactory dysfunction in Parkinson’s disease. Neurosignals.

[66] Driver-Duckley E., Adler C.H., Hentz J.G., Dugger B.N., Shill H.A., Caviness J.N., Sabbagh M.N., Beach T.G., Arizona Parkinson Disease Consortium (2014). Olfactory dysfunction in incidental Lewy body disease and Parkinson’s disease. Parkinsonism Relat. Disord..

[67] Alves J., Petrosyan A., Magalhaes R. (2014). Olfactory disfunction in dementia. World J. Clin. Cases.

[68] Bohnen N.I., Gedela S., Herath P. (2008). Selective hyposmia in Parkinson disease: Association with hippocampal dopamine activity. Neurosci. Lett..

[69] Mega M.S., Cummings J.L., Fiorello T., Gornbein J. (1996). The spectrum of behavioral changes in Alzheimer’s Disease. Neurol.

[70] Menza M.A., Robertson-Hoffman D.E., Bonapace A.S. (1993). Parkinson’s disease and anxiety: Comorbidity with depression. Biol. Psychiatry.

[71] Lee I., Hunsaker M.R., Kesner R.P. (2005). The Role of Hippocampal Subregions in Detecting Spatial Novelty. Behav. Neurosci..

[72] Saab B.J., Georgiou J., Nath A., Lee F.J.S., Wang M., Michalon A., Liu F., Mansuy I.M., Roder J.C. (2009). NCS-1 in the Dentate Gyrus Promotes Exploration, Synaptic Plasticity, and Rapid Acquisition of Spatial Memory. Neuron.

[73] Lever C., Burton S., O’Keefe J. (2006). Rearing on hind legs, environmental novelty, and the hippocampal formation. Rev. Neurosci..

[74] Scott J.C. (2007). Neurocognitive effects of methamphetamine: A critical review and meta-analysis. Neuropsychol. Rev..

[75] Tirelli E., Jodogne C. (1990). Dopamine-GABAergic mechanisms of rearing and locomotion in infant and weanling mice. Psychobiology.

[76] Weinshenker D., Warren S.T. (2008). Fragile dopamine. Nature.

[77] Russell K.H., Giordano M., Sanberg P.R. (1987). Amphetamine-induced on- and off-wall rearing in adult laboratory rats. Pharmacol. Biochem. Behav..

[78] Menon M.K., Clark W.G. (1979). GABA-ergic drugs block the locomotor stimulant effects of 1,3-dimethyl-5-aminoadamantane (D-145). Neuropharmacology.

[79] Scheel-Kruger J., Christensen A.V., Arnt J. (1978). Muscimol differentially facilitates stereotypy but antagonizes motility induced by dopaminergic drugs: A complex GABA-dopamine interaction. Life Sci..

[80] Callaghan R.C., Cunningham J.K., Sykes J., Kish S.J. (2012). Increased risk of Parkinson’s disease in individuals hospitalized with conditions related to the use of methamphetamine or other amphetamine-type drugs. Drug Alcohol Depend..

[81] Rudnicki S.A., Archer R.L., Labib B.T. (2007). Motor neuron disease in methamphetamine abusers. Amyotroph. Lateral Scler..

[82] NIH-National Institute on Drug Abuse (2000). Methamphetamine Abuse Linked to Long-Term Damage to Brain Cells.

[83] Walf A.A., Frye C.A. (2007). The use of the elevated plus maze as an assay of anxiety-related behavior in rodents. Nat. Protoc..

[84] Marcondes F.K., Miguel K.J., Melo L.L., Spadari-Bratfisch R.C. (2001). Estrous cycle influences the response of female rats in the elevated plus-maze test. Physiol. Behav..

[85] Hard E., Engel J., Larsson K., Musi B. (1985). Effect of Diazepam, Apomorphine and Haloperidol on the audiogenic immobility reaction and on the open field behavior. Psychopharmacology.

[86] Hard E., Ahlenius S., Engel J. (1983). Effects of neonatal treatment with 5,7-dihydroxytryptamine or 6-hydroxydopamine on the ontogenetic development of the audiogenic immobility reaction in the rat. Psychopharmacology.

[87] Stopford C.L., Thompson J.C., Neary D., Richardson A.M., Snowden J.S. (2012). Working memory, attention, and executive function in Alzheimer’s disease and frontotemporal dementia. Cortex.

[88] Hodges H. (1996). Maze procedures: The radial-arm and water maze compared. Brain Res. Cogn. Brain Res..

[89] Clark R.E., Broadbent N.J., Squire L.R. (2007). The hippocampus and spatial memory: Findings with a novel modification of the water maze. J. Neurosci..

[90] Buenz E.J., Howe C.L. (2007). Beta-methylamino-alanine (BMAA) injures hippocampal neurons in vivo. Neurotoxicology.

[91] Terry A.V., Buccafusco J.J. (2009). Chapter 13: Spatial Navigation (Water Maze) Tasks in Methods of Behaviour Analysis in Neuroscience.

[92] Bromley-Brits K., Deng Y., Song W. (2011). Morris Water Maze Test for Learning and Memory Deficits in Alzheimer’s Disease Model Mice. J. Vis. Exp..

[93] Sutherland R.J., Hoesing J.M., Vogt B.A., Gabriel M. (1993). Posterior cingulate cortex and spa- tial memory: A microlimnology analysis. Neurobiology of Cingulate Cortex and Limbic Thalamus: A Comprehensive Handbook.

[94] Voorhees C.V., Williams M.T. (2014). Assessing spatial learning and memory in rodents. ILAR.

[95] Santiago M., Matarredona E.R., Machado A., Cano J. (2006). Acute perfusion of BMAA in the rat’s striatum by in vivo microdialysis. Toxicol. Lett..

[96] Lindstrom H., Luthman J., Mouton P., Spencer P., Olson L. (1990). Plant-derived neurotoxic amino acid (beta-*N*-oxalylamino-l-alanine and beta-*N*-methylamino-l-alanine): Effects on central monoamine neurons. J. Neurochem..

[97] Herlenius E., Langercrantz H. (2004). Development of neurotransmitter systems during critical periods. Exp. Neurol..

[98] Clancy B., Kersh B., Hyde J., Darlington R.B., Anand K.J.S., Finlay B.L. (2007). Web-based method for translating neurodevelopment from laboratory species to humans. Neuroinformatics.

[99] Banack S.A., Metcalf J.S., Spáčil Z., Downing T.G., Downing S., Long A., Nunn P.B., Cox P.A. (2011). Distinguishing the cyanobacterial neurotoxin β-*N*-methylamino-l-alanine (BMAA) from other diamino acids. Toxicon.

[100] Lamprea M.R., Cardenas F.P., Setem J., Morato S. (2008). Thigmotactic responses in an open field. Braz. J. Med. Biol. Res..

[101] Bailey K.R., Crawley J.N. (2009). Anxiety-related disorders in Mice.

[102] Conn P.M. (1993). Paradigms for the Study of Behaviour. Methods in Neuroscience.

[103] Roos M.W., Ericsson A., Berg M., Sperber G.O., Sjoquist M., Meyerson B.J. (2003). Functional evaluation of cerebral microembolization in the rat. Brain Res..

[104] Olton D.S., Samuelson R.J. (1976). Remembrance of places passed: Spatial memory in rats. J. Exp. Psychol..

[105] Wenk G.L., Gerfen C., Holmes A., Sibley D., Skolnick P., Wray S. (2004). Assessment of spatial memory using the radial arm maze and Morris Water Maze. Current Protocols in Neuroscience.

[106] Penley S.C., Gaudet C.M., Threlkeld S.W. (2013). Use of an Eight-arm Radial Water Maze to Assess Working and Reference Memory Following Neonatal Brain Injury. J. Vis. Exp..

